# Targeted multiplex proteomics for molecular prescreening and biomarker discovery in metastatic colorectal cancer

**DOI:** 10.1038/s41598-019-49867-7

**Published:** 2019-09-19

**Authors:** Garazi Serna, Fiorella Ruiz-Pace, Fabiola Cecchi, Roberta Fasani, Jose Jimenez, Sheeno Thyparambil, Stefania Landolfi, Elena Elez, Ana Vivancos, Todd Hembrough, Josep Tabernero, Rodrigo Dienstmann, Paolo Nuciforo

**Affiliations:** 1 Molecular Oncology Group, Vall d’Hebron Institute of Oncology, Barcelona, Spain; 2 Oncology Data Science Group, Vall d’Hebron Institute of Oncology, Barcelona, Spain; 3 NantOmics, LLC, Rockville, MD, USA; 4 Pathology Department, Vall d’Hebron University Hospital, CIBERONC, Barcelona, Spain; 5 Medical Oncology Department, Vall d’Hebron University Hospital, Barcelona, Spain; 6 Genomics Group, Vall d’Hebron Institute of Oncology, Barcelona, Spain

**Keywords:** Colon cancer, Colon cancer, Predictive markers, Molecular medicine

## Abstract

Protein biomarkers are widely used in cancer diagnosis, prognosis, and prediction of treatment response. Here we introduce the use of targeted multiplex proteomics (TMP) as a tool to simultaneously measure a panel of 54 proteins involved in oncogenic, tumour suppression, drug metabolism and resistance, in patients with metastatic colorectal cancer (mCRC). TMP provided valuable diagnostic information by unmasking an occult neuroendocrine differentiation and identifying a misclassified case based on abnormal proteins phenotype. No significant differences in protein levels between unpaired primary and metastatic samples were observed. Four proteins were found differentially expressed in *KRAS*-mutant as compared to wild-type tumours (overexpressed in mutant: KRAS, EGFR; overexpressed in wild-type: TOPO1, TOP2A). Survival analyses revealed the association between mesothelin expression and poor overall survival, whereas lack of PTEN protein expression associated with lower progression-free survival with anti-EGFR-based therapy in the first-line setting for patients with *RAS* wild-type tumour. Finally, outlier analysis identified putative targetable proteins in 65% of patients lacking a targetable genomic alteration. Our data show that TMP constitutes a promising, novel molecular prescreening tool in mCRC to identify protein expression alterations that may impact on patient outcomes and more precisely guide patient eligibility to clinical trials with novel targeted experimental therapies.

## Introduction

Simultaneous and accurate measurement of many proteins in experimental samples is very important in descriptive and predictive biological research, including comprehensive proteomic surveys, protein network studies, validation of genomic alterations and clinical biomarker development.

The accepted ‘gold standard’ for protein measurement is immunoassay, which uses either one (as in immunohistochemistry or IHC) or two (competitive immunoassays) antigen-specific antibodies. Antibody-antigen binding can be measured across multiple matrices such as in fluids, on the surface of cells, within cells or tissues and in organs. The most widely used platform for tissue-based biomarker analyses is IHC, which facilitates the qualitative expression of proteins while preserving tissue architecture. Despite being a relatively easy and inexpensive approach, IHC has its limitations including reproducibility issues and very poor multiplexing capabilities. It is at best a semiquantitative method and is minimally effective for comprehensive analyses.

Targeted proteomics using selected reaction monitoring mass spectrometry (SRM-MS) has emerged as a promising and highly sensitive method for the quantification of multiple proteins within the same tissue sample in an antibody-free setting^1–3^. However, its application to formalin -fixed paraffin -embedded (FFPE) tissues, which represent the standard preservation method for tumour samples analysed in the clinic, has been hampered by incomplete solubilization of samples^4,5^.

In the present study, we used the Liquid Tissue® proteomic method to reverse formalin-induced crosslinks allowing complete solubilization of all proteins in the sample for the precise quantification^6,7^. The reliability of this approach for the analysis of proteins in patient tumour tissues has been previously demonstrated^8–13^. Our targeted panel of proteins including oncogenic, tumour suppression, drug metabolism, and drug resistance markers, among others were measured in FFPE tissues from 50 patients with mCRC treated at our institution and whose tumours were genomically-profiled as a prescreening strategy for clinical trial recruitment. We performed an exploratory correlative analysis of protein levels with clinicopathological and genomic markers, investigated their association with the patient outcome – including response to standard chemotherapies and combinations with anti-EGFR agents – and assessed their potential as predictive biomarkers for novel targeted experimental therapies currently under investigation.

## Results

### Exploratory expression analysis of targeted multiplex proteomics in mCRC

Clinicopathological characteristics of patients included in our study are summarized in Table 1 (Supplementary Table 1 for individual data). All patients consented to participate in a molecular prescreening programme that included targeted next-generation sequencing and *MET* amplification status by fluorescence *in situ* hybridization to identify potentially targetable alterations. FFPE blocks were retrieved from the pathology archive of the Vall d’Hebron University Hospital.

**Table 1:** Patients’ characteristics.

**Age, median (range) years**	57.9 (28–73)
**Sex**	
Male	32 (64%)
Female	18 (36%)
**Location**	
Left colon	31 (62%)
Right colon	7 (14%)
Rectum	7 (14%)
NA	5 (10%)
**Metastatic sites at diagnosis**	
0	17 (34%)
>0	33 (66%)
**RAS status**	
*KRAS* Mutated	20 (40%)
*NRAS* Mutated	1 (2%)
*RAS* Wild-type	29 (58%)
**PIK3CA status**	
Mutated	6 (12%)
Wild-type	44 (88%)
**Tissue**	
Primary	26 (52%)
Liver metastasis	24 (48%)
**First-line chemotherapy**	
Irinotecan + 5FU	14 (28%)
Oxaliplatin + 5FU	16 (32%)
Oxaliplatin + 5FU + anti-EGFR	8 (16%)
Irinotecan + 5FU + anti-EGFR	9 (18%)
Other	3 (6%)
**Second-line chemotherapy**	
Irinotecan + 5FU	22 (44%)
Oxaliplatin + 5FU	5 (10%)
Oxaliplatin + 5FU + anti-EGFR	2 (4%)
Irinotecan + 5FU + anti-EGFR	13 (26%)
anti-EGFR alone	1 (2%)
Not given	7 (14%)
**Survival, median (95% confidence interval) months**	
Survival metastatic setting	44.3 (40–58)
Time to progression first-line	8.3 (7–11)
Time to progression second-line	6.9 (6–9)

**Figure 1:**
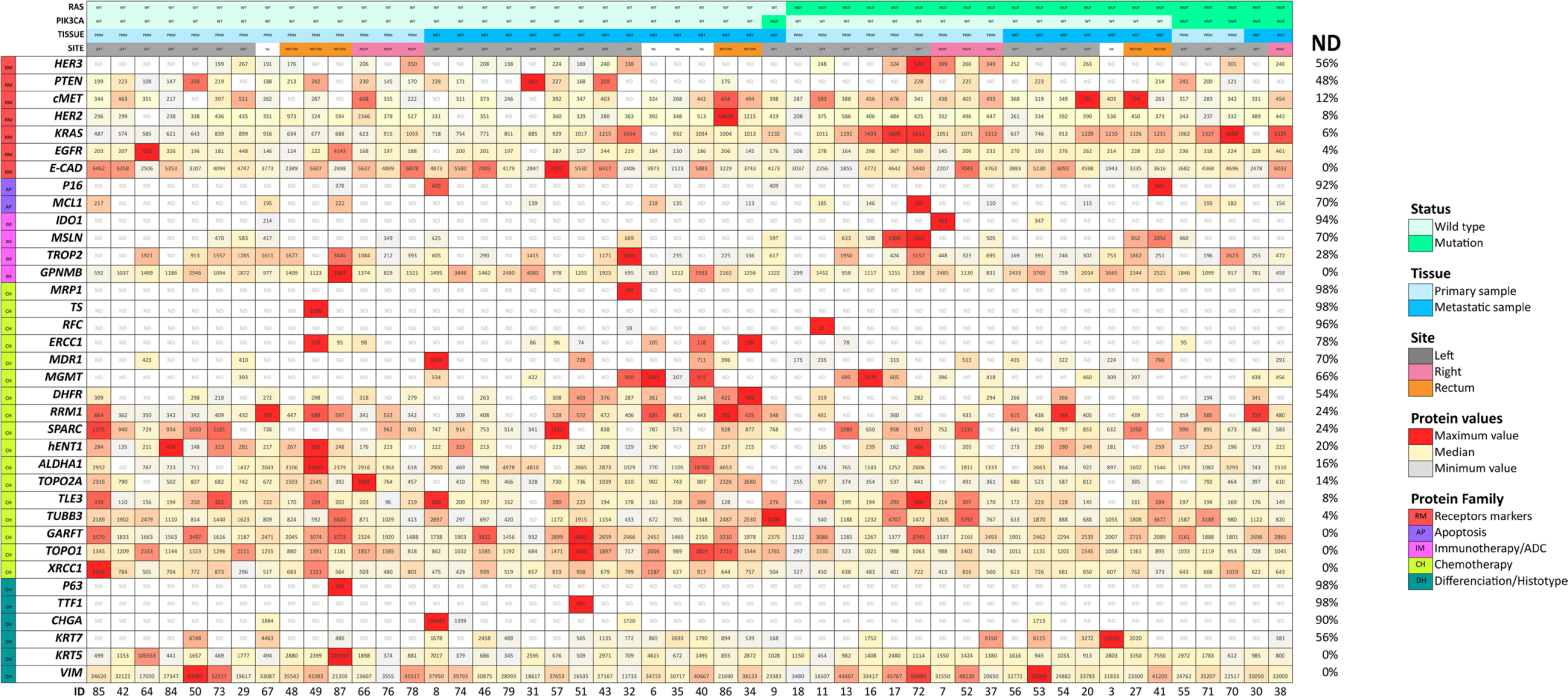
Targeted multiplex proteomics heatmap. Individual samples are plotted in columns (case IDs are indicated at the bottom of each column). Proteins are plotted in rows. Each cell shows the protein level in amol/μg. For each protein, levels of expression are shown on a colour scale from the lowest (light grey) to the highest (red) value. ND indicates non-detectable levels. Percentage of ND for each protein is shown in the rightmost column. *RAS* and *PIK3CA* status (MUT, mutation; WT, wild type), tissue (PRIM, primary sample; MET, metastasis), and site are indicated; NA: not applicable.

To obtain a pure population of tumour cells without the surrounding microenvironment, we performed laser-capture microdissection. Isolated tumour cells were then solubilized to tryptic peptides using Liquid Tissue® technology as previously described^10^. All samples were qualified as evaluable according to the expression levels of the two housekeeping proteins actin and tubulin. We based the selection of the target proteins included in our TMP panel on a literature search for predictive biomarkers of response to targeted/chemo/immunotherapy and also included differentiation proteins with diagnostic value. Among 54 target proteins analysed, 18 (33%) were below the limit of detection of SRM-MS (non-detectable) (Supplementary Table 2). FGF receptors (FGFR1-3), IGF1R, HGF, and PDL1 were among non-detectable proteins. Thirty-six proteins (67%) were detectable in at least one sample. Levels of expression (amol/μg) of detectable proteins for each individual patient are shown in Fig. 1 as heatmaps (see also supplementary figure 1 for aggregated data). The threshold for the definition of high protein expression varied by protein. We used predefined criteria when supporting literature was available: ≥4,000 amol/μg for EGFR^14^, ≥750 amol/μg for HER2^11,13^ and ≥1500 amol/μg for cMET^12^. These thresholds were shown to be predictive of protein overexpression and/or gene amplification detected by standard IHC and fluorescence *in situ* hybridization. For the remaining ones, SRM-MS levels above the upper 95% confidence interval of the mean were defined as high expression (Supplementary Table 3).

The TMP panel included 7 proteins routinely used in diagnostic IHC including cytokeratins (KRT5 and KRT7), markers of stratified epithelia (P63), mesenchymal (Vimentin) and neuroendocrine (CHGA, SYP) differentiation, and primary tumour origin (TTF1). Analysis of differentiation markers showed a lack of expression of P63 and TTF1 in all but 2 cases (4%), consistent with the colorectal origin of our samples. One tumour (ID-87) with P63 expression also showed very high levels of EGFR and KRT5, highly suggestive of squamous cell differentiation. Upon histopathological review, the case turned out to be an anal squamous cell carcinoma and, therefore, excluded from further analyses. The only case in our dataset expressing TTF1 was a liver metastasis (ID-51). Thyroid transcription factor 1 (TTF1) has been considered a highly sensitive and specific marker for primary lung adenocarcinoma. As it is not usually expressed in CRC, TTF1 is used for the differential diagnosis of metastatic adenocarcinomas of colorectal versus lung origin^15,16^. The subsequent IHC workup on ID-51 showed strong TTF1 nuclear positivity, KRT20 positivity and KRT7 negativity, a profile pointing to colorectal origin, which was supported by clinical assessment. Five tumours showed detectable levels of CHGA, a marker of neuroendocrine differentiation. IHC analyses confirmed the presence of CHGA positive cells in otherwise conventional adenocarcinomas of the colon, and outlined an occult neuroendocrine differentiation in one case (ID-08) with very high levels (10-times higher than those of the lowest detectable case) (Supplementary Figure 2A).

Since samples from both primary tumours (n = 25) and metastatic disease (n = 24) were included in our cohort, we investigated differences in protein expression levels according to the tissue of origin. Overall, no significant difference between unpaired primary and metastatic lesions was observed (BH adjusted Mann-Whitney p > 0.05 for all comparisons) (Supplementary Table 4). When dichotomized (detectable vs non-detectable), KRT7 was found expressed in 19% of primaries and 71% of metastatic samples (Pearson’s Chi-squared p = 0.003). Confirmatory IHC analyses showed that KRT7 expression prevailed in tumour buds present in both primary and metastatic samples as has been previously described^17^. Worthy of note, entrapped normal epithelial cells from the lung were the main contributor of KRT7 expression in sample ID-53 (Supplementary Figure 2B).

### Protein expression in *RAS*-mutated and wild-type tumours

Understanding that *RAS* mutation is a validated stratification factor in mCRC, we then examined differences in proteins levels in *RAS*-mutated (n = 21, 43%) versus wild-type cases (n = 28, 57%). KRAS and EGFR expression levels were higher in the *RAS*-mutated subgroup as compared to wild-type (BH adjusted Mann-Whitney p = 0.023 for both comparisons). Median KRAS expression levels were 1192 amol/μg (IQR 1011-1327) and 867 amol/μg (IQR 640-1006) for *RAS*-mutated and wild-type tumours, respectively (no differences were found according to codon mutated). Median EGFR expression levels were 232 amol/μg (IQR 210-299) in *RAS*-mutated and 192 amol/μg (IQR 165-206) in wild-type tumours. On the other hand, TOPO1 and TOP2A were significantly underexpressed in *RAS*-mutated tumours (BH adjusted Mann-Whitney p = 0.023 for both comparisons). Median TOPO1 expression levels were 1408 amol/μg (IQR 1116-1893) and 1033 amol/μg (IQR 953-1131) for *RAS* wild-type and mutated tumours, respectively. TOP2A levels were 753 amol/μg (IQR 492-936) in *RAS* wild-type and 441 amol/μg (IQT 354-587) in mutated tumours (Supplementary Table 5).

### Targeted multiplex proteomics identifies MSLN as a prognostic marker in mCRC

**Figure 2:**
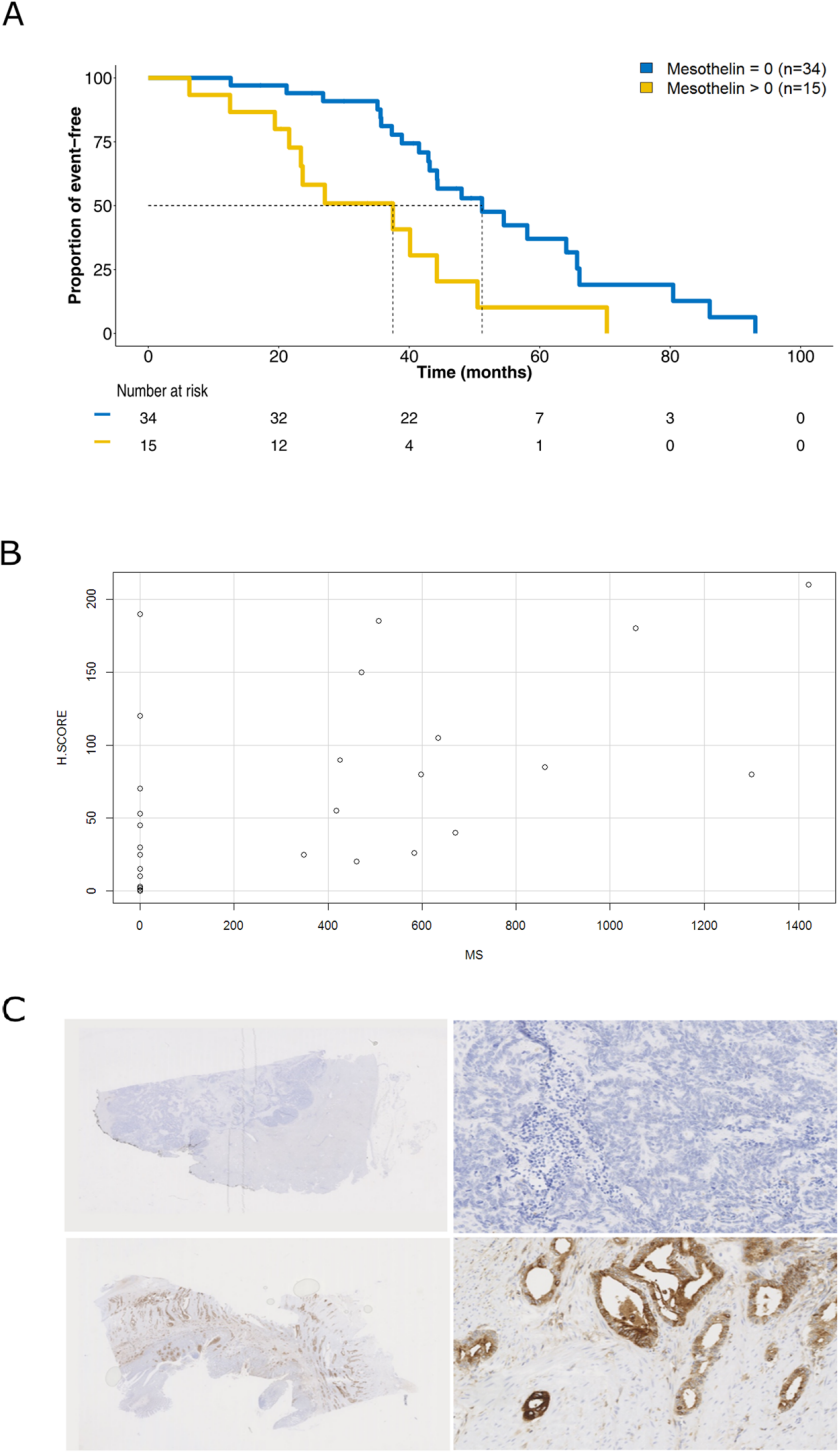
Mesothelin (MSLN) expression in colorectal cancer. (**A**) Kaplan-Meier analysis of overall survival in the metastatic setting according to MSLN expression levels quantified by mass spectrometry (MS). (**B**) Correlation between MS (x-axis, amol/ug) and immunohistochemistry (y-axis, H-score). Pearson correlation coefficient, 95% confidence interval and p-value are indicated. (**C**) Representative immunohistochemistry staining of a MS MSLN-negative (upper panel; ID-79, liver metastasis, H-score = 0) and –positive (lower panel; ID-72, primary CRC, H-score = 210) cases. Digital magnification: 0.5 × (left) and 20 × (right).

We assessed the prognostic value of protein expression levels in terms of overall survival (OS) in the metastatic setting. In univariate analysis, after multiple testing adjustment, only MSLN levels associated with outcome (Log-Rank test, p = 0.004; Fig. 2A, Supplementary Table 6). We found MSLN expression (SRM-MS > 0) in 15 out of 49 cases (30%) of mCRC by SRM-MS. In a multivariate analysis, MSLN remained an independent factor predicting worse OS in the metastatic setting (Cox proportional-hazard test, p = 0.04; Supplementary Table 7). Interestingly, MSLN expression levels were comparable with those found in one control mesothelioma included in the analysis (748.5 amol/μg, data not shown).

To confirm proteomics-driven results and determine which cell type was expressing MSLN in the context of CRC, we compared expression results generated by SRM-MS with standard IHC. All but 3 samples previously profiled with SRM-MS could be analysed with IHC. A strong correlation between SRM-MS and IHC as continuous MSLN expression values was found (Spearman’s rank correlation coefficient 0.669, p < 0.0001, Fig. 2B). When dichotomizing for positive versus negative cases, we observed an overall concurrence of 83% between the two approaches (Fisher test, p = 0.0001). MSLN expression was observed mainly at the tumour cell membrane but also in the cytoplasmic compartment (Fig. 2C). There was no association between MSLN expression by either SRM-MS or IHC and tumour stage at diagnosis (III vs IV), sample origin (primary versus metastasis), primary tumour location, and RAS mutation status (Supplementary Figure 3A). High levels of MSLN were associated with worse OS independently of the methodology used (Supplementary Figure 3B).

### Protein expression as a predictor of clinical benefit with targeted and standard therapies

A total of 23 *RAS* wild-type patients received an anti-EGFR therapy as first or second line of treatment. Lack of PTEN protein expression significantly associated with a high risk of progression in the first-line anti-EGFR setting, with a time to progression of 4.2 months versus 9.4 months in patients whose tumours expressed PTEN (HR = 3.7, CI95% 1.1–13.1, p = 0.03). No association between other protein expression levels (including EGFR) and response to EGFR targeted therapy was found.

A panel of 19 proteins involved in chemosensitivity or chemoresistance was analysed in our TMP assay. Among those, thymidylate synthase (TS), excision repair cross-complementing group 1 (ERCC1), and Topoisomerase 1 (TOPO1) have been previously studied as putative biomarkers of response to 5-FU, oxaliplatin, and irinotecan, respectively^18–22^. In our dataset, TS was detectable in a single case (2%) whereas ERCC1 and TOPO1 were expressed in 22% and 100% of mCRC analysed (6% and 28% at high levels, respectively). No association between ERCC1 and benefit from treatment with oxaliplatin-based chemotherapy was found (n = 28 eligible patients, Pearson’s Chi-squared p-value = 0.62). Similarly, we did not find a significant association between clinical benefit from irinotecan-based therapy and TOPO1 expression when dichotomized at the high-level cut-off (n = 44 eligible patients, Pearson’s Chi-squared test p = 1).

### Protein expression signposts treatment selection in early clinical trials

One of the major goals of our study was to explore whether multiplex proteomic analysis could complement current genomic molecular prescreening by identifying abnormal proteins expression in patients without any targetable genomic alteration. Out of 49 patients, 6 (12%) had *PIK3CA* exon 9 or 20 mutations (all but one case with a co-existing *KRAS* mutation) eligible for combination therapy with PI3K inhibitors at our institution.

**Figure 3:**
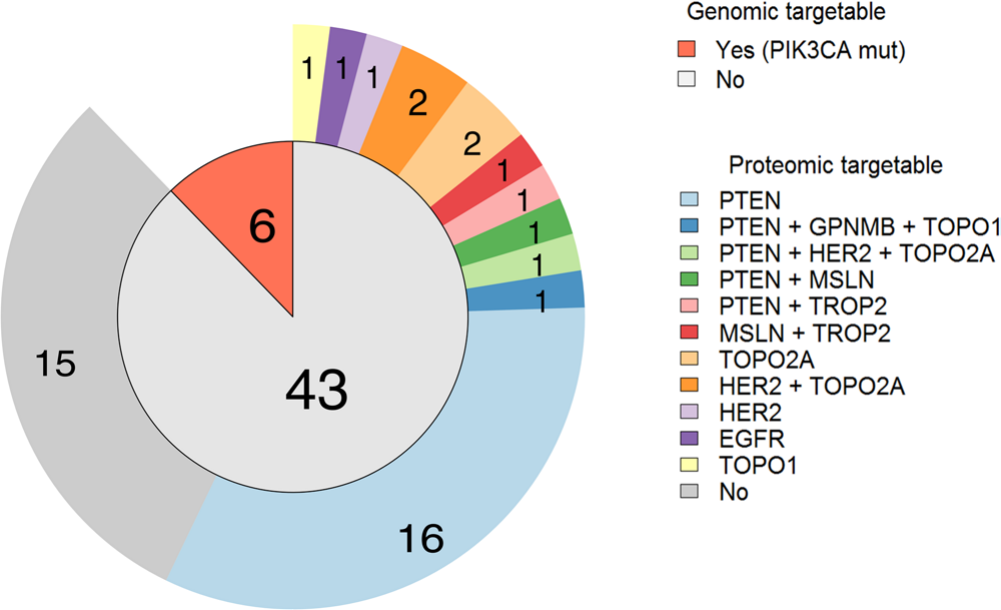
Distribution of genomic targetable alterations (inner circle). Detailed proteomic targetable alterations for non-genomics targetable samples (outer ring).

In the remaining 43 patients without targetable genomic alteration, SRM-MS identified 20 patients (47%) without PTEN expression (non-detectable), enrichment criteria for PI3K pathway inhibitors. In addition, 1 tumour had very high expression of EGFR (7635 amol/μg) and 4 harboured high expression levels of HER2 (confirmed by standard IHC, Supplementary Figure 2C). This could guide enrollment in clinical trials with novel HER family inhibitors. No patient had high cMET protein expression (in line with the lack of *MET* amplification by fluorescence *in situ* hybridization in our cohort). With regards to other proteins measured in our TMP panel, in the absence of a validated threshold for overexpression by SRM-MS, we conducted Robust regression and Outlier removal (ROUT) analysis^23^ (false discovery rate of 1%). A total of 31 outliers (proteins expressed at very high levels) across 12 proteins were identified in 24 individual patients (Supplementary Figure 4). Twenty-nine (93.5%) outliers were found in 21 patients without any targetable genomic alteration. Among these, 12 could be potentially used to guide the selection of an investigational antibody-drug conjugate treatment (GPNMB, MSLN, and TROP2) or refine a chemotherapy strategy (TOPO1 and TOP2A). Overall, 28 out of 43 patients (65%) whose tumours lacked a genomic match to a clinical trial were eligible for investigational drugs as a result of TMP analysis (PTEN, EGFR, HER2, GPNMB, MSLN, TROP2, TOPO1 or TOP2A as emerging positive predictive markers) (Fig. 3).

## Discussion

Therapies guided by tumour molecular profiling have shown significant clinical benefit in treating patients with advanced cancer. In mCRC, examples of emerging markers include *BRAFV600E* mutations^24^, HER2 overexpression/amplification^25,26^, and *MET* amplification^27^. However, in aggregate, these predictors are positive in less than 10% of mCRC population, indicating the need for more informative molecular screening approaches. Similarly, the decision to administer standard chemotherapies in the metastatic setting (e.g. FOLFOX or FOLFIRI) remains empirical and does not rely on tumour biomarkers, which could be revealed by a novel multiplatform profiling strategy like ours.

To the best of our knowledge, this is the first study to explore the potential impact of quantitative targeted proteomics in the context of precision oncology in mCRC. A comprehensive molecular profiling of CRC samples has been previously conducted with a multiplatform approach that includes sequencing, IHC, fluorescence *in situ* hybridization and chromogenic *in situ* hybridization to investigate targetable biomarker aberrations^28^. Authors found major differences in the expression of selected proteins across different metastatic sites, reflecting inter-metastatic tumour heterogeneity, although the limited overlap in term of proteins analyzed (only 15 in total) and the different methodology used for protein expression (IHC vs SRM-MS in our study) hampered any comparative analysis.

In our study, we investigated quantitative protein biomarker profiles of mCRC and integrated the results obtained with available clinical, pathological and genomic data towards advancing insights into predictive and prognostic markers. Our TMP panel included proteins involved in oncogenic, tumour suppression, drug metabolism and resistance as well as tumour differentiation markers that could aid in the standard diagnostic decision-making processes as well as the selection of novel targeted drugs and immunotherapies (antibody-drug conjugates or ADC).

The major impact of the use of TMP in mCRC may translate in more precisely matching patients to experimental therapies. Recruitment rates in early clinical trials based on genomic markers or targeted IHC in metastatic CRC does not exceed 15%^29,30^. We expect that proteomics-guided drug development will expand treatment options for patients who are eligible to participate in early phase clinical studies, particularly in view of the increasing array of ADCs and immunotherapeutic approaches, thus repurposing proteomics as an important contender in precision oncology.

Until validated thresholds for individual biomarkers become available, outlier expression may be a valuable marker for testing novel experimental therapies in the metastatic setting as well as an enrichment strategy for clinical trials. In our present study, we found 29 outliers in 21 patients without any targetable genomic alteration, 12 of which are potentially useful in more precisely guiding the selection of an investigational ADC therapy (GPNMB, MSLN, and TROP2) or refining a chemotherapy strategy (TOPO1 and TOP2A).

With regards to novel prognostic markers and therapeutic targets, our TMP analysis identified MSLN expression in 30% of metastatic CRC. MSLN is a cell-surface glycoprotein whose expression in normal human tissues is restricted to mesothelial cells. Given that it is highly expressed by many solid tumours (mesotheliomas, pancreatic, gastric and ovarian cancers among others), it represents an attractive target for ADC development^31^. In mCRC, the value of MSLN expression has not been thoroughly investigated. The prognostic association we found between MSLN and poor prognosis has been previously reported in the literature with both tumour and plasma MSLN levels in early-stage CRC^32,33^ thus strengthening the role of this protein in CRC biology. This supports its investigation as a therapeutic target in this yet unexplored tumour indication.

We also investigated the value of TMP in patient selection for standard-of-care targeted agents. We recognize the difficulties in identifying predictive markers for anti-EGFR and standard therapies in our cohort since these therapies are administered in combination regimens and the small sample size that together do not allow for the proper study of interactions among different markers and therapies. We did, however, identify an association between a lack of PTEN protein expression and shorter time to progression in the first-line setting with chemotherapy plus anti-EGFR in *RAS* wild-type patients. Previous retrospective studies^34–37^ showed that this association could well be linked to a prognostic effect of PTEN in this subset of patients^38,39^ as opposed to a predictive value^40^.

Exploratory analysis of proteomics data allowed us to assess the value of complementary data provided by this approach in the context of the molecular diagnostic evaluation of mCRC patients. First, TMP may assist in refining conventional histopathological diagnosis, identifying misdiagnosed cases, and revealing uncommon differentiation that may help clinicians to prioritize alternative treatment regimens. For example, we could confirm an occult neuroendocrine differentiation in one case as well as an atypical proteomics profile which facilitated the identification of a patient sample erroneously submitted as colorectal adenocarcinoma for molecular analyses. Second, primary tumour molecular profiling is generally used for treatment decision even in the metastatic setting where cancer may have acquired new alterations or lost those originally present during carcinogenesis. Our analysis failed to reveal significant differences in protein expression levels between unmatched primary and metastatic samples (continuous protein levels), thus supporting current profiling strategies at least for the targets included in our panel. Finally, comprehensive proteomics analysis may also expose biological differences in clinically or molecularly-defined groups. We examined differences in the biomarker profiles based on *RAS* mutation status. KRAS protein levels were significantly higher in *RAS* mutant vs wild-type CRC, independently of the mutation type. This is not surprising as *KRAS* mutations occurring in codons 12 and 13 of exon 2 induce stabilization of the protein in a constitutive activation state which, in theory, could also facilitate the detection of higher levels of the total protein in mutant tumours as compared to wild-type^41^. We also found that TOPO1 and TOP2A expression levels were lower in *RAS*-mutated tumours, an association not previously reported in the literature, and that reinforces differences in the biology of these tumours. Higher TOPO1 expression in *RAS* wild-type may favour irinotecan over oxaliplatin in this molecular subgroup, and many trials are assessing irinotecan plus anti-EGFR therapy re-challenge after an initial response in *RAS* wild-type mCRC. Similarly, high TOP2A levels may suggest an increased sensitivity to topoisomerase II inhibitors^42,43^ in *RAS* wild-type CRC and prompt the exploration of agents not routinely considered as standard-of-care. These results certainly merit further consideration in future clinical trials.

In conclusion, this study opens the door to the application of targeted multiplex proteomics in CRC with a potential impact on patient stratification for precision medicine. Our results reinforce the inter-tumour heterogeneity of CRC with unique protein alterations in individual cases that can guide enrolment in early clinical trials and co-development of biomarkers and drugs. We expect that an expanded TMP panel with proteins that represent targets for ADCs and immune checkpoints may bring novel insights on potential combinatorial immunotherapeutic strategies.

## Methods

### Patient selection

Samples of histologically confirmed invasive CRC diagnosed at Vall d’Hebron University Hospital (Barcelona, Spain) were retrospectively identified by a study pathologist. All patients included in the study underwent a molecular prescreening between 2013 and 2014 which included an amplicon sequencing analysis of a panel of 59 genes^44^ and MET amplification status by fluorescence *in situ* hybridization. The protocol of this study was approved by the Vall d’Hebron University Hospital Ethical Committee (PR(AG)147-2009) and all methods were performed following relevant guidelines and regulations. Informed consent was obtained from all patients. Clinicopathological characteristics are shown in Table 1 and Supplementary Table 1.

### SRM-MS analysis

Proteins were quantitated by SRM-MS as previously described^7,10^. Briefly, tissue sections (10 μM) were cut from FFPE blocks, placed onto Director® microdissection slides, deparaffinized and stained with haematoxylin. Tumour areas were marked by a board-certified pathologist. Microdissection of the marked area was performed using a laser microdissector (Molecular Machines, Germany). A total area of 12 mm^2^ containing approximately 45,000 malignant cells was microdissected from each tumour and transferred directly into the cap of 0.65 ml tube containing 20 µl of 100% acetonitrile. Acetonitrile was removed by SpeedVac centrifugation at 35 °C for 6 min. The dried dissection pellet was stored at −20 °C. Peptides were extracted from the pellet using the Liquid Tissue® technology as per manufacturer’s instructions (Expression Pathology, NantOmics, Rockville, MD, available for purchase at NantOmics). The Liquid Tissue® protocol involves heating the tumour tissue at 95 °C for 90 minutes using the Liquid Tissue® buffer, followed by trypsin digestion for 18 hours at 37 °C. The resulting peptide concentration is measured by the microBCA assay. Heavy labelled internal standards are added along with buffer A (0.1% formic acid). 10 µl of this mixture is injected into the mass spectrometer (Thermo TSQ Quantiva) and a targeted list of peptides (see Supplementary Table 2 for targets included in the panel) are quantitated using selected reaction monitoring. On-column injection results in 1 µg (~4000 cells) of solubilized tissue and 5 fmol of an internal standard. Normalization was conducted across all samples based on the total amount of protein. Data analysis was carried out using Pinnacle (Optys Tech, MA). For QC purposes, the levels of actin or tubulin had to be greater than 298 amol/µg or 28 amol/µg respectively in all samples. Instrumental analyses were performed on TSQ series (Vantage or Quantiva) of triple quadrupole mass spectrometer (Thermo Scientific, San Jose, CA).

Assay development and QC was along the lines of our previously published work^12^. Briefly, tryptic digestion of recombinant proteins, fixed cell lines or tissue was conducted. The peptides from recombinant proteins were first injected into the triple quadrupole mass spectrometer to identify the peptide characteristics (retention time, transition ion ratios, and area under the curve). This information was used to identify the peptides from fixed cell lines or FFPE tissue samples. The top two (based on AUC, reproducibility) unique peptides from FFPE or fixed cell lines were chosen for further development. Unlabelled and labelled synthetic peptides of the top two unique peptides were used to assess the analytical performance (LoD, LoQ, precision, and carryover). Eventually, the peptide that had the best analytical performance was used to quantitate the protein of interest.

### Immunohistochemistry

The following primary monoclonal antibodies were used: anti-chromogranin A (CHGA), anti-cytokeratin 7 (KRT7), anti-HER2, and anti-mesothelin (MSLN). Before cutting, paraffin blocks were cooled to −10 °C and 3 µm sections were cut with a microtome. To ensure tissue straightening, sections were floated on distilled water at 43 °C. Cut tissues were collected on positively charged Superfrost glass slides and slides were dried overnight at 37 °C. Immunohistochemical stainings of HER2, KRT7 and CHGA were performed using a Benchmark ULTRA autostainer (Ventana Medical Systems, Tucson AZ). The slides were heated in the instrument and deparaffinized with EZ prep solution (Ventana Medical Systems, Tucson AZ). Heat-induced antigen retrieval was executed using Cell Conditioning 1 (CC1; Ventana Medical Systems, Tucson AZ) for 36 min at 95 °C. Then, the primary antibody was applied as indicated in Supplementary Table 8. Reactions were detected using the UltraView Universal DAB Detection kit (#760–500; Ventana Medical Systems, Tucson AZ). Finally, the slides were counterstained with Haematoxylin II and Bluing Reagent (Ventana Medical Systems, Tucson AZ) and mounted with xylene-based mounting medium.

Staining of MSLN was carried out using the rabbit Envision-kit (#K4003; DAKO/Agilent, Santa Clara, CA). Briefly, the sections were deparaffinized with xylene, dehydrated with decreasing ethanol baths and hydrated with distilled water and then heated in the PTLink for 20 minutes at 95 °C with Dako Target Retrieval Solution pH 9 (#S2367; DAKO/Agilent, Santa Clara, CA), diluted 1:10 with distilled water. Endogenous peroxidase activities were inactivated with Dako Envision Flex Peroxidase Blocking Reagent for 15 min at room temperature and then protein block (#X0909; DAKO/Agilent, Santa Clara, CA) was applied for 30 minutes at room temperature. Samples were then incubated with rabbit anti-human MSLN monoclonal antibody (clone SP74) at room temperature for 1 hour (dilution 1:2 with Dako REAL Ab Diluent #S2022; DAKO/Agilent, Santa Clara, CA). Flex HRP was then applied for 30 min at room temperature, followed by incubation with Dako DAB detection solution FLEX-DAB for 5 minutes at room temperature. Slides were counterstained with Merck Haematoxylin Harris (dilution 1:4) for 2 min, dehydrated and mounted with Xylol based mounting medium. IHC stained slides were scanned using NanoZoomer 2.0-HT (Hamamatsu Photonics, Japan) and subsequently, the digital images were scored by a pathologist blinded to the MSLN SRM-MS results. For the assessment of MSLN, a semi-quantitative approach was used in which H-scores were generated by multiplying the staining intensity (0 = no staining, 1 = weak, 2 = moderate, 3 = strong) by the percentage of positive cells (0-100%). MSLN staining was considered positive when at least 15% of the cells showed moderate (2+) to strong (3+) intensity.

### Statistical analysis

A descriptive analysis of the variables included in the study was performed. Continuous variables were expressed as median and IQR, and categorical variables were expressed as absolute values and percentages. Comparison of continuous variables was performed with non-parametric Mann-Witney test (two groups) or Kruskal-Wallis test (more than two) with adjustment for multiple testing according to Benjamini and Hochberg (BH) method. Spearman’s rank correlation coefficient was used to study the association between two continuous variables. For the univariate analysis of categorical variables, we used the Chi-squared test or Fisher exact test if cell frequencies were below 5. To assess their association with clinical benefit with standard chemotherapies, we classified patients as responders when the time to progression (calculated from the date of treatment initiation to progression or death) in the first-line or second-line treatments exceeded their respective medians (8.3 and 6.9 months, respectively).

Overall survival (OS) in the metastatic setting as an interval from the diagnosis of metastasis to death. Survival analysis was calculated using the Kaplan–Meier method and the log-rank test was used for statistical comparison. Hazard ratios (HRs) and associated 95% confidence interval were assessed using Cox proportional-hazard. ROUT analysis with a false discovery rate of 1% was conducted to identify outliers. Statistical significance was accepted at the conventional two-sided p < 0.05 threshold. The data analyses were carried out using R version 3.3.3 statistical software package.

## Supplementary information

Supplementary information

Supplementary Table 1

## Data Availability

All data generated or analysed during this study are included in this published article (and its Supplementary Information files).

## References

[1] Addona TA (2009). Multi-site assessment of the precision and reproducibility of multiple reaction monitoring–based measurements of proteins in plasma. Nat. Biotechnol..

[2] Nilsson T (2010). Mass spectrometry in high-throughput proteomics: ready for the big time. Nat. Methods.

[3] Rudnick PA (2010). Performance Metrics for Liquid Chromatography-Tandem Mass Spectrometry Systems in Proteomics Analyses. Mol. Cell. Proteomics.

[4] Prieto DA (2005). Liquid Tissue: proteomic profiling of formalin-fixed tissues. Biotechniques.

[5] Bateman NW (2011). Differential Proteomic Analysis of Late-Stage and Recurrent Breast Cancer from Formalin-Fixed Paraffin-Embedded Tissues. J. Proteome Res..

[6] Hood BL (2005). Proteomic Analysis of Formalin-fixed Prostate Cancer Tissue. Mol. Cell. Proteomics.

[7] Hembrough T (2013). Application of Selected Reaction Monitoring for Multiplex Quantification of Clinically Validated Biomarkers in Formalin-Fixed, Paraffin-Embedded Tumor Tissue. J. Mol. Diagnostics.

[8] Huang SK (2009). LC/MS-Based Quantitative Proteomic Analysis of Paraffin-Embedded Archival Melanomas Reveals Potential Proteomic Biomarkers Associated with Metastasis. PLoS One.

[9] DeSouza LV (2010). mTRAQ-based quantification of potential endometrial carcinoma biomarkers from archived formalin-fixed paraffin-embedded tissues. Proteomics.

[10] Hembrough T (2012). Selected Reaction Monitoring (SRM) Analysis of Epidermal Growth Factor Receptor (EGFR) in Formalin Fixed Tumor Tissue. Clin. Proteomics.

[11] Nuciforo P (2016). High HER2 protein levels correlate with increased survival in breast cancer patients treated with anti-HER2 therapy. Mol. Oncol..

[12] Catenacci DVT (2014). Absolute Quantitation of Met Using Mass Spectrometry for Clinical Application: Assay Precision, Stability, and Correlation with MET Gene Amplification in FFPE Tumor Tissue. PLoS One.

[13] Catenacci DVT (2016). Mass-spectrometry-based quantitation of Her2 in gastroesophageal tumor tissue: comparison to IHC and FISH. Gastric Cancer.

[14] Maron SB (2018). Targeted Therapies for Targeted Populations: Anti-EGFR Treatment for *EGFR* -Amplified Gastroesophageal Adenocarcinoma. Cancer Discov..

[15] Moldvay J (2004). The role of TTF-1 in differentiating primary and metastatic lung adenocarcinomas. Pathol. Oncol. Res..

[16] Compérat E (2005). Variable sensitivity and specificity of TTF-1 antibodies in lung metastatic adenocarcinoma of colorectal origin. Mod. Pathol..

[17] Harbaum L (2011). Keratin 7 expression in colorectal cancer - freak of nature or significant finding?. Histopathology.

[18] Choueiri MB (2015). ERCC1 and TS Expression as Prognostic and Predictive Biomarkers in Metastatic Colon Cancer. PLoS One.

[19] Grimminger PP (2012). TS and ERCC-1 mRNA expressions and clinical outcome in patients with metastatic colon cancer in CONFIRM-1 and −2 clinical trials. Pharmacogenomics J..

[20] Li P (2013). ERCC1, defective mismatch repair status as predictive biomarkers of survival for stage III colon cancer patients receiving oxaliplatin-based adjuvant chemotherapy. Br. J. Cancer.

[21] Shirota Y (2001). *ERCC1* and Thymidylate Synthase mRNA Levels Predict Survival for Colorectal Cancer Patients Receiving Combination Oxaliplatin and Fluorouracil Chemotherapy. J. Clin. Oncol..

[22] Li Z (2014). Predictive value of APE1, BRCA1, ERCC1 and TUBB3 expression in patients with advanced non-small cell lung cancer (NSCLC) receiving first-line platinum–paclitaxel chemotherapy. Cancer Chemother. Pharmacol..

[23] Motulsky Harvey J, Brown Ronald E (2006). BMC Bioinformatics.

[24] Hong DS (2016). Phase IB Study of Vemurafenib in Combination with Irinotecan and Cetuximab in Patients with Metastatic Colorectal Cancer with BRAFV600E Mutation. Cancer Discov..

[25] Sartore-Bianchi A (2016). Dual-targeted therapy with trastuzumab and lapatinib in treatment-refractory, KRAS codon 12/13 wild-type, HER2-positive metastatic colorectal cancer (HERACLES): a proof-of-concept, multicentre, open-label, phase 2 trial. Lancet Oncol..

[26] Hainsworth JD (2018). Targeted Therapy for Advanced Solid Tumors on the Basis of Molecular Profiles: Results From MyPathway, an Open-Label, Phase IIa Multiple Basket Study. J. Clin. Oncol..

[27] Poulsen TT (2017). Sym015: A Highly Efficacious Antibody Mixture against *MET* -Amplified Tumors. Clin. Cancer Res..

[28] El-Deiry WS (2015). Molecular profiling of 6,892 colorectal cancer samples suggests different possible treatment options specific to metastatic sites. Cancer Biol. Ther..

[29] Dienstmann R (2017). Analysis of mutant allele fractions in driver genes in colorectal cancer - biological and clinical insights. Mol. Oncol..

[30] Overman MJ (2016). Utility of a molecular prescreening program in advanced colorectal cancer for enrollment on biomarker-selected clinical trials. Ann. Oncol..

[31] Morello A, Sadelain M, Adusumilli PS (2016). Mesothelin-Targeted CARs: Driving T Cells to Solid Tumors. Cancer Discov..

[32] Foda AAM, El-Hawary AK, Hamed H (2016). Aberrant Expression of Calretinin, D2–40 and Mesothelin in Mucinous and Non-Mucinous Colorectal Carcinomas and Relation to Clinicopathological Features and Prognosis. Pathol. Oncol. Res..

[33] Li S (2017). Plasma Mesothelin as a Novel Diagnostic and Prognostic Biomarker in Colorectal Cancer. J. Cancer.

[34] Frattini M (2007). PTEN loss of expression predicts cetuximab efficacy in metastatic colorectal cancer patients. Br. J. Cancer.

[35] Loupakis F (2009). PTEN Expression and KRAS Mutations on Primary Tumors and Metastases in the Prediction of Benefit From Cetuximab Plus Irinotecan for Patients With Metastatic Colorectal Cancer. J. Clin. Oncol..

[36] Sartore-Bianchi A (2009). *PIK3CA* Mutations in Colorectal Cancer Are Associated with Clinical Resistance to EGFR-Targeted Monoclonal Antibodies. Cancer Res..

[37] Razis E (2008). Potential value of PTEN in predicting cetuximab response in colorectal cancer: An exploratory study. BMC Cancer.

[38] Sawai H (2008). Loss of PTEN expression is associated with colorectal cancer liver metastasis and poor patient survival. BMC Gastroenterol..

[39] Laurent-Puig P (2009). Analysis of *PTEN*, *BRAF*, and *EGFR* Status in Determining Benefit From Cetuximab Therapy in Wild-Type *KRAS* Metastatic Colon Cancer. J. Clin. Oncol..

[40] Personeni N (2015). FOLFIRI and Cetuximab Every Second Week for First-Line Treatment of KRAS Wild-Type Metastatic Colorectal Cancer According to Phosphatase and Tensin Homolog Expression: A Phase II Study. Clin. Colorectal Cancer.

[41] Takashima A, Faller DV (2013). Targeting the RAS oncogene. Expert Opin. Ther. Targets.

[42] Desmedt C (2011). Multifactorial Approach to Predicting Resistance to Anthracyclines. J. Clin. Oncol..

[43] Bartlett JMS (2015). Predicting Anthracycline Benefit: *TOP2A* and CEP17—Not Only but Also. J. Clin. Oncol..

[44] Thress KS (2015). Acquired EGFR C797S mutation mediates resistance to AZD9291 in non–small cell lung cancer harboring EGFR T790M. Nat. Med..

