# Supplementary material for: Targeted multiplex proteomics for molecular prescreening and biomarker discovery in metastatic colorectal cancer
Source: Sci Rep. 2019 Sep 19;9:13568. doi: 10.1038/s41598-019-49867-7 (PMC6753065; doi:10.1038/s41598-019-49867-7)
Supplement: Supplementary file 1 — Supplementary information [file 41598_2019_49867_MOESM1_ESM.pdf]

Supplementary material

Targeted multiplex proteomics for molecular prescreening and biomarker discovery in metastatic colorectal cancer

Garazi Serna[1], Fiorella Ruiz-Pace[2], Fabiola Cecchi[3], Roberta Fasani[1], Jose Jimenez[1], Sheeno Thyparambil[3], Stefania Landolfi[4], Elena Elez[5], Ana Vivancos[6], Todd Hembrough[3], Josep Tabernero[5], Rodrigo Dienstmann[2], Paolo Nuciforo[1*]

[1] Molecular Oncology Group, Vall d'Hebron Instutute of Oncology, Barcelona, Spain
[2] Oncology Data Science Group, Vall d'Hebron Instutute of Oncology, Barcelona, Spain
[3] NantOmincs, LLC
[4] Pathology Department, Vall d'Hebron University Hospital, CIBERONC, Barcelona, Spain
[5] Medical Oncology Department, Vall d'Hebron University Hospital, Barcelona, Spain
[6] Genomics Group, Vall d'Hebron Instutute of Oncology, Barcelona, Spain

Corresponding author:

Paolo Nuciforo, MD PhD
Principal Investigator, Molecular Oncology Group
Vall d'Hebron University Hospital
Vall d'Hebron Institute of Oncology (VHIO)
C/ Natzaret, 115-117
08035 Barcelona, SPAIN
Tel: + 34 93 254 34 50 Ext 8626
pnuciforo@vhio.net

Supplementary tables and figures legend

Supplementary table 1. Colorectal cancer dataset included in the study: individual patient clinicopathological features and SRM-MS protein levels. Non-detectable proteins are indicated as “0”.

Supplementary table 2. List of proteins quantified by SRM-MS. Targets detectable in at least one samples are indicated.

Supplementary table 3. Descriptive statistics of detectable proteins in colorectal cancer samples. Percentage of samples classified as “High” is indicated in the bottom row.

Supplementary table 4. Comparison of protein expression levels between unmatched primary and metastatic samples. P-values calculated using non-parametric Kruskal-Wallis test with adjustment for multiple testing according to Benjamini and Hochberg (BH) method are indicated.

Supplementary table 5. Comparison of protein expression levels between RAS-mutated versus RAS-wild type samples. P-values calculated using non-parametric Kruskal-Wallis test with adjustment for multiple testing according to Benjamini and Hochberg (BH) method are indicated.

Supplementary table 6. Association between selected proteins and overall survival. Proteins were classified as low versus high as described in the main text. Log-rank test was used for statistical comparison. P-values are indicated.

Supplementary table 7. Multivariable model of overall survival including mesothelin together with known prognostic factor in the metastatic setting. Cox proportional-hazard model was used to obtain hazard ratios (HRs) with 95% CIs.

Supplementary table 8. Primary antibody sources, dilutions and incubation time and temperatures used in this study.

Supplementary Figure 1. Distribution of protein expressions across samples. In the bottom panel dark shadow represents high expression. (A) Receptors and downstream markers. Differentiation and histotypes markers. Apoptosis, immunotherapy and ADC proteins. (B) Chemotherapy markers. The definition of high protein expression varied by protein: ≥ 4,000 amol/µg for EGFR13, ≥750 amol/µg for HER2 and ≥1500 amol/µg for cMET. For the remaining proteins, SRM-MS levels above the upper 95% confidence interval of the mean were defined as high expression.

Supplementary Figure 2. Representative immunohistochemistry images of CHGA (A), CK7 (B) and HER2 (C). Sample ID (white) and target protein levels by SRM-MS (colored) are shown on the top right side of each image. ND, not detectable.

Supplementary Figure 3. Association between MSLN levels and clinicopathological features. (A) Data are displayed as box plots. Protein levels

by immunohistochemistry (Hscore, left plot) and mass spectrometry (MS, right plot) are shown on the y-axis for each subgroup. P-values (calculated using Wilcoxon test for two variables and Kruskal test for three variable) are indicated. (B) Kaplan-Meier overall survival curves according to MSLN expression levels determined by immunohistochemistry (left side) and mass spectrometry (right side). Hazard ratios together with 95% CI and p-values are shown.

Supplementary Figure 4. ROUT outlier analysis. The twenty-four patients with outlier expression in at least one protein are shown together with the affected target. Presence of mutations in APC, TP53, KRAS, and PIK3CA are indicated by the orange boxes. Outlier protein values (expressed in amol/µg) are shown in the greed boxes for each individual sample.

Supplementary table 2

Family	Protein	SRM-MS
Differenciation/Histotype	**KRT5**	detactable in at least one sample
	**KRT7**	detactable in at least one sample
	**Vimentin**	detactable in at least one sample
	**CHGA**	detactable in at least one sample
	**SYP**	not detectable
	**P63**	detactable in at least one sample
	**TTF1**	detactable in at least one sample
HER	**EGFR**	detactable in at least one sample
	**Her2**	detactable in at least one sample
	**Her3**	detactable in at least one sample
	**Her4**	not detectable
Protein targets	**cMet**	detactable in at least one sample
	**HGF**	not detectable
	**IGF1R**	not detectable
	**PTEN**	detactable in at least one sample
	**KRAS**	detactable in at least one sample
	**ALK**	not detectable
	**ROS1**	not detectable
	**FRalpha**	not detectable
	**AR**	not detectable
	**ER**	not detectable
	**FGFR1**	not detectable
	**FGFR2**	not detectable
	**FGFR3**	not detectable
	**AURKA**	not detectable
	**Axl**	not detectable
	**E-CAD**	detactable in at least one sample
Immuno/ADC	**PDL1**	not detectable
	**MSLN**	detactable in at least one sample
	**IDO1**	detactable in at least one sample
	**GPNMB**	detactable in at least one sample
	**TROP2**	detactable in at least one sample
Apoptosis	**MCL1**	detactable in at least one sample
	**MDM2**	not detectable
	**P16**	detactable in at least one sample
Chemotherapy	**SPARC**	detactable in at least one sample
	**RRM1**	detactable in at least one sample
	**hENT1**	detactable in at least one sample
	**TOPO1**	detactable in at least one sample
	**TOP2A**	detactable in at least one sample
	**ERCC1**	detactable in at least one sample
	**MGMT**	detactable in at least one sample
	**XRCC1**	detactable in at least one sample
	**MRP1**	detactable in at least one sample
	**MDR1**	detactable in at least one sample
	**TUBB3**	detactable in at least one sample
	**DHFR**	detactable in at least one sample
	**RFC**	detactable in at least one sample
	**GRAFT**	detactable in at least one sample
	**TLE3**	detactable in at least one sample
	**ALDHA1**	detactable in at least one sample
	**FPGS**	not detectable
	**TYMS**	not detectable
	**TS**	detactable in at least one sample
Housekeeping	**Actin**	detactable in at least one sample
	**Tubulin**	detactable in at least one sample

Supplementary table 3

	HER			Protein markers				Differenciation/Histotype						Apoptosis		Immunotherapy/ADC			
	**EGFR**	**Her2**	**Her3**	**cMet**	**PTEN**	**KRAS**	**E-CAD**	**KRT5**	**KRT7**	**Vimentin**	**CHGA**	**P63**	**TTF1**	**MCL1**	**P16**	**GPNMB**	**IDO1**	**MSLN**	**TROP2**
**Number of detectable**	48	46	22	44	26	47	50	50	22	50	5	1	1	15	4	50	3	15	36
**Number of non detectable**	2	4	28	6	24	3	0	0	28	0	45	49	49	35	46	0	47	35	14
**Total number of samples**	50	50	50	50	50	50	50	50	50	50	50	50	50	50	50	50	50	50	50
**Minimum**	106.2	207.9	168.8	217	107.9	487	1855	344.6	168.3	3480	1399	533.7	561	109.5	377.5	298.6	214.5	348.9	168.9
**25% Percentile**	184.9	332.3	204.3	317.7	171.1	745.5	3181.2	683.6	558.4	23104	1556.1	533.7	561	134.5	385.3	977.9	214.5	459.8	293.1
**Median**	207.9	383.9	249.9	370.3	216.6	1003.5	4482.9	1151.5	1715	32061	1719.5	533.7	561	181.8	506.9	1315	346.7	583.3	610.9
**75% Percentile**	268.1	485.2	327.1	455.6	228.4	1209.5	5586.7	2508.8	3569.6	38526	59275	533.7	561	216.6	632.8	2317.3	493.2	861.7	1597.1
**Maximum**	7635	14530	519.7	757	281.1	1669	8706.7	229167	17333	65050	116667	533.7	561	355.4	642	9386.7	493.2	1422	5365
**Mean**	465.6	767.3	269.6	396.6	203.9	995.9	4426.9	8314.9	2943.7	31910	24676	533.7	561	178.4	508.3	1829.3	351.5	683.6	1079.8
**Std. Deviation**	1201.2	2100.7	83.9	125.9	42.9	301.8	1584.2	35206.8	3909.3	13112	51425	0	0	62.7	134.5	1515.3	139.4	329.8	1132
**Std. Error of Mean**	173.4	309.7	17.9	18.9	8.4	44.0	224.0	4978.9	833.5	1854.3	22998	0	0	16.2	67.2	214.3	80.5	85.2	188.7
**Lower 95% CI of mean**	116.8	143.4	232.4	358.4	186.6	907.3	3976.7	-1690.6	1210.4	28184	-39176	0	0	143.7	294.3	1398.7	5.2	500.9	696.8
**Upper 95% CI of mean**	814.4	1391.1	306.8	434.9	221.2	1084.5	4877.1	18320.5	4677	35636	88529	0	0	213.1	722.3	2259.9	697.8	866.2	1462.8
**% above 95% CI (HIGH)**	4%	8%	12%	0%	24%	30%	36%	4%	8%	30%	2%	2%	2%	8%	0%	26%	0%	6%	20%

Supplementary table 3 (con't)

	Chemotherapy																	Housekeeping	
	ALDHA1	DHFR	ERCC1	GRAFT	hENT1	MDR1	MGMT	MRP1	RFC	RRM1	SPARC	TLE3	TOPO1	TOP2A	TS	TUBB3	XRCC1	Actin	Tubulin
**Number of detectable**	42	23	11	50	40	15	17	1	2	38	38	46	50	43	1	48	50	50	50
**Number of non detectable**	8	27	39	0	10	35	33	49	48	12	12	4	0	7	49	2	0	0	0
**Total number of samples**	50	50	50	50	50	50	50	50	50	50	50	50	50	50	50	50	50	50	50
**Minimum**	468.7	194	74.4	931.7	129.1	175.4	207.3	445.2	58.7	309.2	341.2	96.4	297.1	255.2	1179.5	297	295.7	165.3	109.2
**25% Percentile**	888.6	271.8	86.2	1785.2	182.7	291.4	394.4	445.2	58.7	394.1	712.5	171.7	987.9	457.5	1179.5	777.2	503.5	452.9	154.3
**Median**	1399.9	298.3	96.2	2226.7	222.5	410	455.7	445.2	69.7	459.4	845.8	199.6	1171	681.8	1179.5	1162.6	646.9	509.4	173.5
**75% Percentile**	2879.8	361.1	118.1	2752.5	257.9	710.5	797.4	445.2	80.7	601.5	959.1	270.9	1555.2	809.5	1179.5	1912	788.1	582.0	204.4
**Maximum**	25817	555.5	130.4	4401.3	423.8	1349.5	1083.3	445.2	80.7	762	1231.2	399.8	3240.3	7465	1179.5	8100	1935.7	1870.3	274.3
**Mean**	2775.1	315.8	100.5	2327.5	232.8	486.1	567.9	445.2	69.7	497.9	838.3	219.2	1320.9	954.5	1179.5	1737.9	689.4	537.8	178.7
**Std. Deviation**	4630.6	76.3	18.7	768.6	69.9	302.0	276.7	0	15.6	135.2	193.8	68.8	579.6	1161.4	0	1592.1	272.7	224.4	33.6
**Std. Error of Mean**	714.5	15.9	5.7	108.7	11.1	77.9	67.1	0	11	21.9	31.4	10.1	81.9	177.1	0	229.8	38.6	31.7	4.7
**Lower 95% CI of mean**	1332.1	282.8	87.9	2109.1	210.4	318.8	425.6	0	-70.1	453.4	774.6	198.7	1156.2	597.1	0	1275.6	611.9	473.9	169.2
**Upper 95% CI of mean**	4218.1	348.8	113.1	2545.9	255.1	653.3	710.2	0	209.5	542.3	901.9	239.6	1485.6	1311.9	0	2200.2	766.9	601.5	188.3
**% above 95% CI (HIGH)**	10%	12%	6%	30%	20%	8%	8%	2%	0%	26%	30%	26%	30%	12%	2%	20%	28%		

Supplementary table 4

	pvals.prim_met.adj
**KRT7**	0.060
**EGFR**	0.634
**Her3**	0.634
**PTEN**	0.634
**MSLN**	0.634
**CHGA**	0.634
**TTF1**	0.634
**P16**	0.634
**SPARC**	0.634
**RRM1**	0.634
**MGMT**	0.634
**MRP1**	0.634
**MDR1**	0.634
**DHFR**	0.634
**GRAFT**	0.634
**TLE3**	0.634
**GPNMB**	0.634
**TS**	0.636
**MCL1**	0.737
**ERCC1**	0.737
**TROP2**	0.737
**TOPO1**	0.785
**XRCC1**	0.785
**TUBB3**	0.835
**IDO1**	0.835
**hENT1**	0.877
**Vimentin**	0.899
**ALDHA1**	0.899
**KRAS**	0.978
**Her2**	0.988
**cMet**	0.988
**E.CAD**	0.988
**KRT5**	0.988
**TOP2A**	0.988
**RFC**	1.000

Supplementary table 5

	pvals.ras.adj
**EGFR**	0.023
**KRAS**	0.023
**TOPO1**	0.023
**TOP2A**	0.023
**PTEN**	0.284
**cMet**	0.330
**XRCC1**	0.330
**ERCC1**	0.340
**DHFR**	0.340
**RRM1**	0.370
**E.CAD**	0.494
**MSLN**	0.494
**Her3**	0.535
**MCL1**	0.535
**MGMT**	0.535
**MDR1**	0.535
**Her2**	0.541
**KRT5**	0.541
**CHGA**	0.541
**TTF1**	0.588
**MRP1**	0.588
**IDO1**	0.588
**TS**	0.588
**TUBB3**	0.595
**ALDHA1**	0.595
**hENT1**	0.601
**KRT7**	0.732
**Vimentin**	0.732
**TROP2**	0.842
**P16**	0.878
**GPNMB**	0.878
**RFC**	0.890
**TLE3**	0.924
**SPARC**	0.946
**GRAFT**	0.984

Supplementary table 6

Protein marker	records	events	median	0.95LCL	0.95UCL	Log-Rank
**MET HIGH**	17	12	48.0	41.5	NA	p=0.144
**MET LOW**	33	24	43.1	35.7	65.7	
**ECAD HIGH**	25	18	44.2	38.9	NA	p=0.400
**ECAD LOW**	25	18	50.1	37.3	66.1	
**MSLN LOW**	35	24	51.2	44.3	66.1	p=0.004
**MSLN HIGH**	15	12	37.5	23.4	NA	
**MCL1 LOW**	35	27	44.3	37.5	65.7	p=0.683
**MCL1 HIGH**	15	9	44.2	41.5	NA	
**MCL1 LOW*1**	30	30	8.0	6.1	12.8	p=0.260
**MCL1 HIGH*1**	15	15	10.1	6.1	11.9	
**MCL1 LOW*2**	30	30	7.4	6.0	10.5	p=0.665
**MCL1 HIGH*2**	15	15	6.4	3.1	15.5	
**MDR1 LOW**	35	26	43.1	35.7	65.7	p=0.255
**MDR1 HIGH**	15	10	51.2	44.3	NA	
**MDR1 LOW*1**	34	34	7.6	6.1	10.7	p=0.673
**MDR1 HIGH*1**	11	11	11.7	7.6	NA	
**MDR1 LOW*2**	34	34	6.9	6.0	9.4	p=0.064
**MDR1 HIGH*2**	11	11	9.3	5.9	NA	
**PTEN LOW**	24	15	48.0	42.9	NA	p=0.805
**PTEN HIGH**	26	21	44.3	35.6	70.3	

Supplementary table 7

Factor	HR	CI95% low	CI95% high	P value
**Mesothelin high vs. low**	2.36	1.04	5.24	0.04
**Liver metastasis vs. no**	1.45	0.55	3.83	0.45
**RAS mut vs. wild-type**	1.17	0.54	2.52	0.69
**Right colon vs. left colon/ rectum**	0.54	0.18	1.62	0.28
**Curative resection of metastasis vs. no**	0.53	0.19	1.45	0.21

Supplementary table 8

Antigen	Clone	Dilution	Manufacturer and reference	Primary antibody incubation time and temperature
**CK7**	SP1	Ready-to-use	Ventana Medical Systems (#790-4462)	20 min Room Temperature
**Chromogranin A**	LK2H10	Ready-to-use	Ventana Medical Systems (#760-2519)	12 min 36ºC
**HER2**	4B5	Ready-to-use	Ventana Medical Systems (#790-2991)	20 min 37ºC

Supplementary figure 1

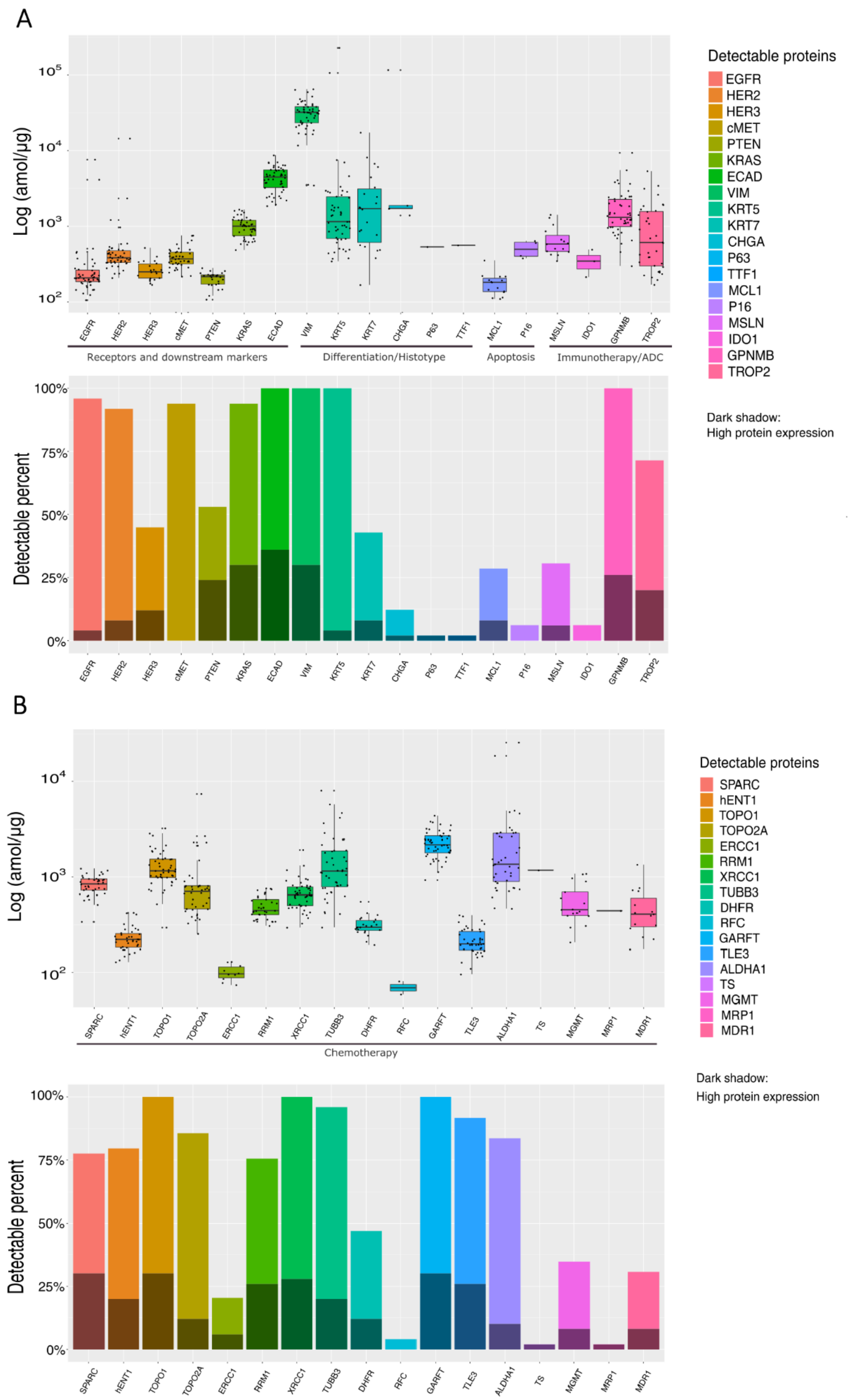

Supplementary figure 2

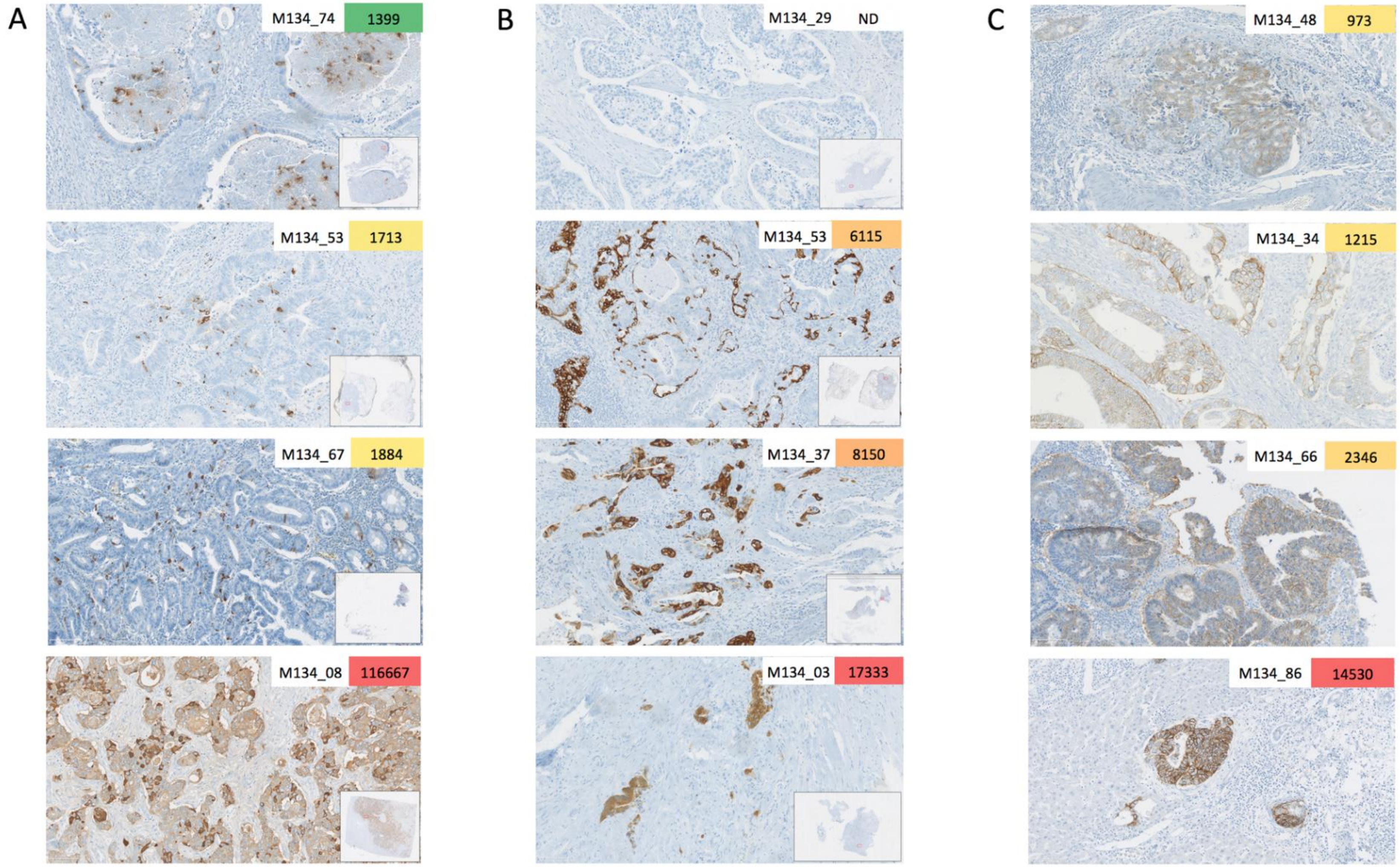

Supplementary Figure 3

A

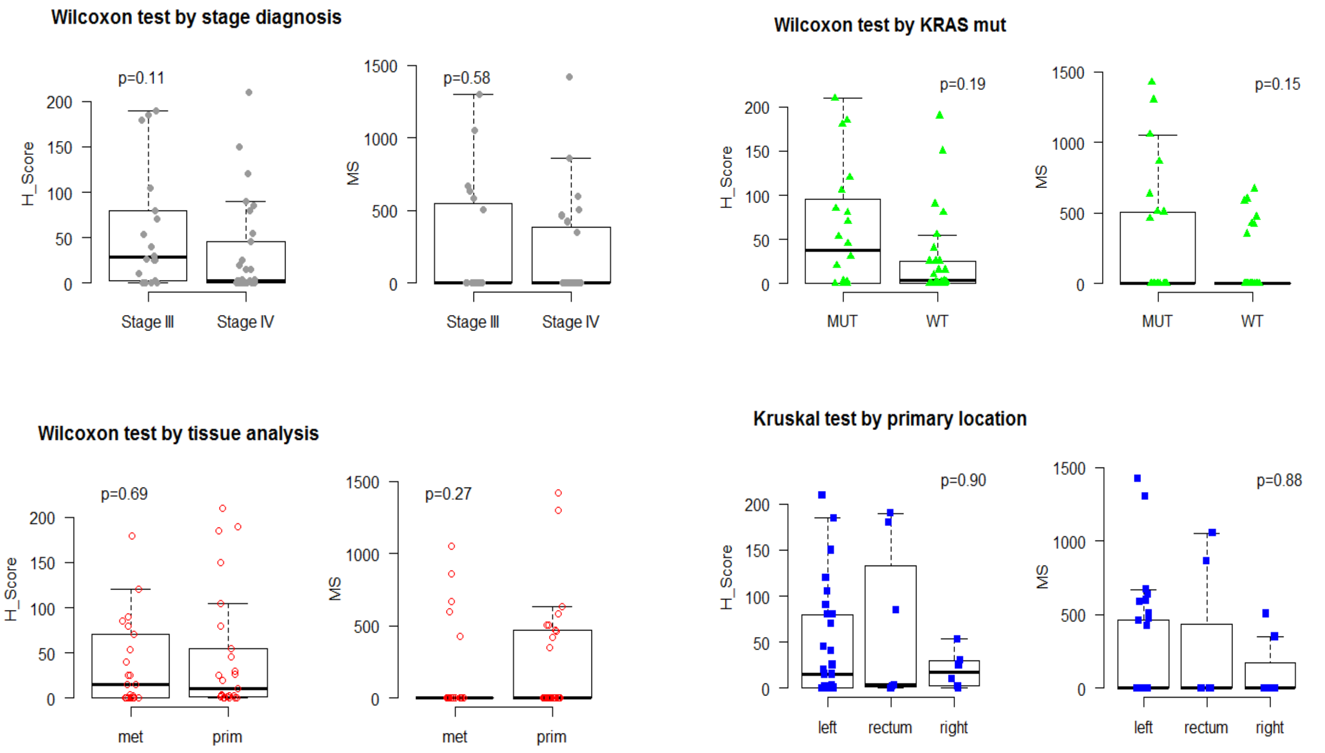

B

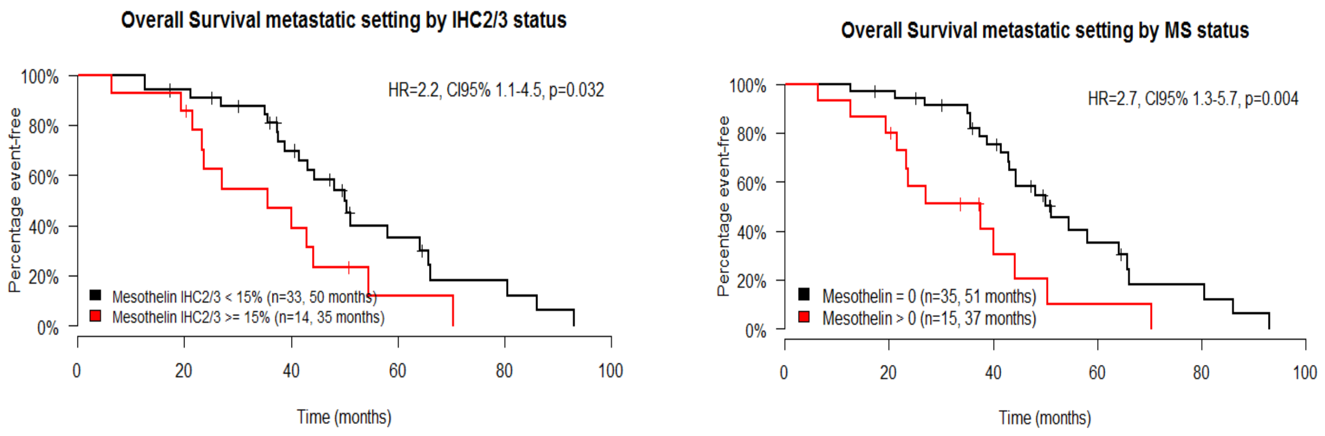

Supplementary figure 4

	03	17	37	41	52	53	38	71	72	66	50	51	48	49	85	09	06	08	29	32	34	40	64	86
APC																								
TP53																								
KRAS																								
PIK3CA																								
KRT5				7550													4615	7017					106933	
KRT7	17333		8150			6115					6748													
CHGA																		116667						
GPNMB																						5533		
MSLN		1300							1422															
TROP2									3157											5365				
ALDHA1														25817								18700		
MDR1																		1350						
TOPO1												3240										2859		
TOP2A										7465				2145	2316						2680			2326
TUBB3		4707		3677	5792			4188								8100								
XRCC1														1321	1936									
